# Non-invasive approaches to monitor EGFR-TKI treatment in non-small-cell lung cancer

**DOI:** 10.1186/s13045-015-0193-6

**Published:** 2015-07-31

**Authors:** Wei Sun, Xun Yuan, Yijun Tian, Hua Wu, Hanxiao Xu, Guoqing Hu, Kongming Wu

**Affiliations:** Department of Oncology, Tongji Hospital, Tongji Medical College, Huazhong University of Science and Technology, 1095 Jiefang Road, Wuhan, 430030 Hubei People’s Republic of China

**Keywords:** Epidermal growth factor receptor, Tyrosine kinase inhibitors, Circulating cell-free tumor DNA, MicroRNAs, Proteomics, Circulating tumor cells, Non-small-cell lung cancer

## Abstract

Tyrosine kinase inhibitors of epidermal growth factor receptor (EGFR-TKIs) are standard treatments for advanced non-small-cell lung cancer (NSCLC) patients harboring activating epidermal growth factor receptor (EGFR) mutations. Nowadays, tumor tissues acquired by surgery or biopsy are the routine materials for EGFR mutation analysis. However, the accessibility of tumor tissues is not always satisfactory in advanced NSCLC. Moreover, a high proportion of NSCLC patients will eventually develop resistance to EGFR-TKIs. Invasive procedures, such as surgery or biopsy, are impractical to be performed repeatedly to assess the evolution of EGFR-TKI resistance. Thus, exploring some convenient and less invasive techniques to monitor EGFR-TKI treatment is urgently needed. Circulating cell-free tumor DNA (ctDNA) has a high degree of specificity to detect EGFR mutations in NSCLC. Besides, ctDNA is capable of monitoring the disease progression during EGFR-TKI treatment. Certain serum microRNAs that correlate with EGFR signaling pathway, such as miR-21 and miR-10b, have been demonstrated to be helpful in evaluating the efficiency of EGFR-TKI therapeutics. A commercialized serum-based proteomic test, named VeriStrat test, has shown an outstanding ability to predict the clinical outcome of NSCLC patients receiving EGFR-TKIs. Analysis of EGFR mutations in circulating tumor cells (CTCs) is feasible, and CTCs represent a promising material to predict EGFR-TKI-treatment efficacy and resistance. These evidences suggested that non-invasive techniques based on serum or plasma samples had a great potential for monitoring EGFR-TKI treatment in NSCLC. In this review, we summarized these non-invasive approaches and considered their possible applications in EGFR-TKI-treatment monitoring.

## Introduction

Lung cancer is the most commonly diagnosed cancer as well as the leading cause of cancer-related deaths [1]. Non-small-cell lung cancer (NSCLC) accounts for approximately 83 % of all lung cancers, and nearly 80 % of NSCLC patients are diagnosed with advanced or distant stages [2]. So far, the common cancer-driver genes identified in NSCLC include the mutant activations of Kirsten rat sarcoma (KRAS), epidermal growth factor receptor (EGFR), proto-oncogene B-Raf (BRAF), and phosphoinositide-3-kinase, catalytic, α polypeptide gene (PIK3CA) [3]. Several other molecular alterations, such as echinoderm microtubule-associated protein-like 4-anaplastic lymphoma kinase (EML4-ALK) fusion [4], Notch signaling activation [5, 6], and dysregulation of the nucleo-cytoplasmic transport of proteins [7], also participate in NSCLC progression. Multiple small-molecule inhibitors directed at these molecular targets have been extensively developed, and some of them are migrating from bench to bedside [4, 7, 8]. However, despite these rapid progresses, tyrosine kinase inhibitors of epidermal growth factor receptor (EGFR-TKIs) are still the most successful example of targeted therapy in NSCLC. Compared with conventional chemotherapy, EGFR-TKIs can achieve prolonged progression-free survival (PFS) with reduced side effects in NSCLC patients harboring activating EGFR mutation [9].

Nowadays, EGFR gene mutations are the standard predictive biomarkers for selecting NSCLC patients to receive EGFR-TKI treatment. The point mutation in exon 21 (L858R) or deletion in exon 19 predicts good response to EGFR-TKIs [10], while the point mutation (T790M) in exon 20 implies resistance to EGFR-TKIs [11]. The routine materials for EGFR mutation analyses are tumor tissues acquired by surgery or biopsy. However, assessment of EGFR mutation status depending on tumor tissues has many limitations. First, the accessibility of tumor tissues is not always satisfactory. Most NSCLC patients are diagnosed with advanced or distant stages. Performing surgery or biopsy to obtain tissues from these patients is often impractical. Only 20 % to 50 % NSCLC patients could provide sufficient tissues to receive molecular analysis even in large well-designed clinical trials [9, 12–15]. Second, a high proportion of NSCLC patients will eventually develop EGFR-TKI resistance [16, 17]. Identifying a molecular mechanism of acquired resistance to EGFR-TKIs and developing relevant targeted drugs are needed for effective second-line treatments [18, 19]. But, assessing treatment resistance in real time by tumor tissues is not feasible as repeated surgery or biopsy is inappropriate in most patients. Furthermore, surgery and biopsy are not without clinical complications. The adverse event rates for thoracic biopsies were reported to be 17.1 % [20]. Considering these limitations, exploring some convenient, economic, and less invasive techniques to monitor EGFR-TKI treatment in NSCLC is urgently needed.

Non-invasive approaches, usually based on plasma or serum samples, showed great potential for monitoring EGFR-TKI treatment in recent years. Circulating cell-free tumor DNA (ctDNA), which is released from tumor cells to circulating blood, can be applied to detect EGFR mutations in NSCLC patients [21–23]. Moreover, the dynamic changes in ctDNA EGFR mutation status could predict the clinical outcome of EGFR-TKI treatment [24]. MicroRNAs (miRNAs) are associated with tumor progression, suggesting their potential applications for treatment monitoring. Several serum miRNAs have been identified to associate with response to EGFR-TKIs in NSCLC [25, 26]. Circulating miRNAs may also act as potential non-invasive biomarkers to monitor EGFR-TKI treatment. Serum proteomics is an emerging science. Analyzing serum for specific proteins by mass spectrometry is becoming an important tool to identify biomarkers in cancers [27]. The VeriStrat test, a commercialized serum-based proteomic test, has emerged as a promising approach to predict the outcome of NSCLC patients receiving EGFR-TKIs [28–31]. Circulating tumor cells (CTCs) can be detected in the blood of many cancer patients and play an important role in metastasis. Several studies have demonstrated that EGFR mutation status in CTCs matches well with that in tumor tissues [32–34]. Isolating DNA from CTCs to receive EGFR mutation analysis may be another effective way to monitor EGFR-TKI treatment.

**Fig. 1:**
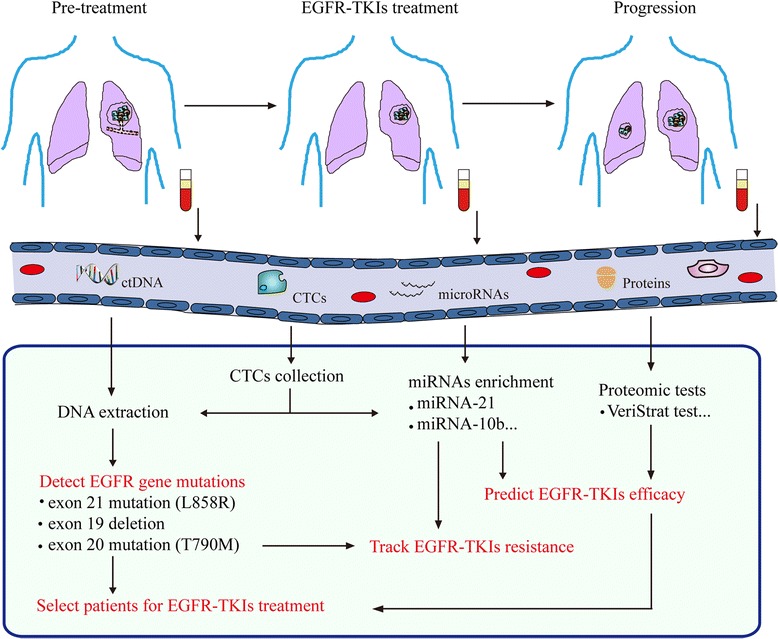
Clinical applications of non-invasive approaches in monitoring EGFR-TKI treatment for NSCLC patients. This schematic diagram depicts the applications of common non-invasive approaches in monitoring EGFR-TKI treatment for NSCLC. Before therapy, ctDNA, CTCs, miRNAs, and proteomic tests help to identify appropriate NSCLC patients to receive EGFR-TKIs. During the course of treatment, ctDNA, CTCs, and miRNAs can be used to monitor EGFR-TKI-treatment response and track EGFR-TKI-treatment resistance. At the time of disease progression, ctDNA, CTCs, and miRNAs reveal the molecular changes related to EGFR-TKI resistance

In summary, serum ctDNA, miRNAs, proteomic biomarkers, and CTCs can be obtained non-invasively, making them ideal substitutes for tumor tissues (Fig. 1). In addition, these liquid biopsies can be performed multiple times and allow for dynamic detection of molecular changes in tumor cells. Hereby, we summarized these non-invasive approaches in this review and considered their possible applications in monitoring EGFR-TKI treatment.

## Circulating cell-free tumor DNA (ctDNA)

Cancer-associated genetic alterations, such as point mutations, deletions, copy number variations, and methylation patterns, can be detected in ctDNA [35, 36]. In NSCLC, ctDNA shows a high diagnostic accuracy for EGFR mutation analyses. Moreover, serum ctDNA analysis could effectively predict the efficacy of EGFR-TKI treatment and identify the molecular changes associated with EGFR-TKI resistance.

### Detection of EGFR gene mutations

When ctDNA is used to analyze gene mutations in NSCLC patients, one vital issue is whether the genetic variation within blood was consistent with tumor tissues. Table 1 summarizes studies which detected EGFR mutations in NSCLC patients using ctDNA. As shown, a wide range of diagnostic accuracy values were reported. The sensitivities for ctDNA ranged from 17.1 % [23] to 100 % [37, 38], while the specificities were from 71.4 % [39] to 100 % [23, 37, 40–47]. Additionally, the concordance rates between ctDNA and tumor tissues showed a big variation, ranging from 27.5 % [23] to 100 % [37]. The large variability among various studies might be caused by different laboratory procedures and techniques utilized for quantification of ctDNA. Besides, the big difference in the numbers of patients might also influence the reported sensitivity and specificity. To address these issues, two recent meta-analyses were performed [48, 49]. Their main purposes were to compare ctDNA with tumor tissues in terms of its diagnostic accuracy for the detection of EGFR mutations. Both of their results revealed that ctDNA had high diagnostic accuracy for the detection of EGFR mutations in NSCLC. The pooled sensitivities for ctDNA in these two meta-analyses were reported to be 67.4 % and 62 %, respectively. And the specificities for ctDNA were 93.5 % and 95.9 %, respectively. These evidences suggested that assessment of EGFR mutations by ctDNA has adequate diagnostic accuracy, especially the high degree of specificity. Thus, ctDNA is a highly specific and effective biomarker for EGFR mutation analyses. Future standard techniques for detecting EGFR mutations in ctDNA might validate its clinical value in NSCLC patients.

**Table 1:** EGFR mutations detected in ctDNA of NSCLC patients

Authors	Case number	Method	Positive (%)	Sensitivity (%)	Specificity (%)	Concordance (%)
Sun H [21]	55	MST-PCR	18.2	NM	NM	NM
Douillard JY [22]	784	ARMS	10.5	65.7	99.8	94.3
Kim HR [23]	40	PNA-PCR	15	17.1	100	27.5
Punnoose EA [37]	24	DxS kits	16.7	100	100	100
Goto K [40]	194	DxS Kits + ARMS	23.7	43	100	66.3
Jian G [81]	56	RT-PCR	23.2	NM	NM	NM
He C [38]	134	ME-PCR	49.3	100	90	94.4
Zhang L [82]	627	ME-PCR	22	NM	NM	NM
Zhao X [83]	111	ME-PCR + sequencing	17.1	35.6	95.5	71.2
Kimura H [39]	27	PCR + sequencing	37	75	71.4	NM
Kim ST [84]	57	PNA-LNA PCR	19.3	66.7	93.3	87.7
Bai H [85]	230	DHPLC	34.3	81.8	89.5	74
Yung TK [41]	35	Digital PCR	43	92	100	NM
Mack PC [42]	49	DxS kits	20.4	66.7	100	NM
Kuang Y [86]	54	ARMS	47	70	85	NM
Kimura H [87]	42	ARMS	16.7	75	97.1	92.9
Kimura H [88]	27	ARMS	48.1	50	85.7	72.7
Brevet M [89]	31	ME-PCR + sequencing	58.1	38.9	84.6	NM
Jiang B [43]	58	ME-PCR + sequencing	24.1	77.8	100	93.1
Wang S [90]	134	ARMS	12.7	22.1	97	59
Jing CW [91]	120	HRM	25.8	64.4	97.3	85
Zhang H [44]	86	MEL	17.4	68.2	100	91.9
Liu X [45]	86	ARMS	31.4	67.5	100	84.9
Xu F [46]	34	ARMS	11.8	50	100	88.2
Huang Z [92]	822	DHPLC	32.7	63.5	84.6	77
Sriram KB [47]	64	ME-PCR	4.7	50	100	95.3
Weber B [50]	196	cobas® EGFR test	11.7	60.7	96.4	91.3

*MST* mutant-specific primers with a Taqman probe, *PCR* polymerase chain reaction, *ARMS* the amplification refractory mutation system, *PNA-PCR* peptide nucleic-acid-mediated PCR, *DxS kits* DxS EGFR Mutation Test Kit (DxS, Manchester, UK), *RT-PCR* real-time PCR, *ME-PCR* mutant-enriched PCR, *PNA-LNA PCR* the peptide nucleic-acid-locked nucleic acid PCR, *DHPLC* denaturing high-performance liquid chromatography, *HRM* high-resolution melting analysis, *MEL* mutant-enriched liquidchip, *NM* not mentioned

### Prediction of EGFR-TKI-treatment efficacy

Regarding the clinical application of ctDNA, another important issue is whether ctDNA is capable of predicting EGFR-TKI-treatment efficacy. Indeed, several studies have analyzed the association between pre-treatment ctDNA EGFR mutation status and clinical outcomes. Goto et al. [40] evaluated EGFR mutations in ctDNA from patients in the IPASS study recruited in Japan (IPASS, The IRESSA Pan-Asia Study). A significant correlation between ctDNA EGFR mutation status and PFS was founded in this study. PFS was significantly longer with gefitinib than carboplatin/paclitaxel in the activating ctDNA EGFR mutation subgroup (hazard ratio (HR), 0.29; 95 % confidence interval (CI), 0.14–0.60). Another large-scale research also demonstrated that EGFR mutation status in ctDNA was a good predictor for PFS after EGFR-TKI therapy (mutant versus wild-type: 10.1 versus 3.7 months, *p* = 0.038) [50]. Recently, the predictive value of the dynamic changes in ctDNA EGFR mutation status during therapy was explored by a prospective study [24]. In this study, patients were randomized to receive six cycles of gemcitabine/platinum plus sequential erlotinib or placebo. Blood samples at baseline, cycle 3, and progression were assessed. In patients with ctDNA-based EGFR mutation-positive status at baseline, those that transformed to EGFR mutation-negative status had better PFS and overall survival (OS) than those with detectable EGFR mutations at cycle 3. These data suggested that dynamic changes of EGFR status in ctDNA were able to predict the benefit of EGFR-TKI treatment. In conclusion, ctDNA EGFR mutation test has a good ability to predict EGFR-TKI-treatment efficacy, and it may be a reliable approach to guide EGFR-TKI treatments for NSCLC patients.

### Tracking molecular changes related to EGFR-TKI resistance

In NSCLC, despite an initial response, resistance to EGFR-TKIs will occur and thereafter tumor progresses. For this reason, identification of the molecular mechanisms of EGFR-TKI resistance is needed. Several mechanisms responsible for EGFR-TKI acquired resistance have been described, including the second T790M mutation of EGFR, amplification of MET or HER2, and mutations of PIK3CA or BRAF [51, 52]. The second T790M mutation of EGFR accounts for half of the TKI-resistant cases, and monitoring T790M mutation is useful for the estimate of EGFR-TKI resistance. Taniguchi et al. performed a study to quantitatively detect the T790M-resistant mutations in ctDNA [53]. In 43.5 % (10/23) of patients who had progressive disease after EGFR-TKI treatment, the T790M mutation in ctDNA was detected. Another study also proved that sequencing of plasma DNA could complement current invasive approaches to identify mutations associated with acquired drug resistance in advanced cancers [36]. This study tracked the genomic evolution during therapy in various kinds of cancer patients by the sequencing of cancer exomes in serial plasma samples. It revealed that the EGFR T790M mutation in plasma could be detected at progression, but not at the start of treatment in NSCLC patients treated with gefitinib. These results suggested that ctDNA was capable of monitoring disease progression during EGFR-TKI therapy.

## MicroRNAs

MicroRNAs (miRNAs) play critical roles in tumor development, including tumor initiation, invasion, and metastasis [54–56]. Several miRNAs that correlate with the EGFR signaling pathway have been demonstrated to mediate EGFR-TKI resistance in NSCLC [57–60]. Moreover, circulating miRNAs are emerging as promising biomarkers and therapeutic targets for many cancers [61–65]. Many studies have shown that changes in circulating miRNAs during treatment are predictive of tumor response [63, 66, 67]. Circulating microRNAs may have the potential to be developed into next-generation biomarkers for cancer screening. In NSCLC patients, certain miRNAs have been detected in the serum samples. These circulating miRNAs are showing their potential in EGFR-TKI-treatment monitoring [25, 26].

Shen et al. performed a study to identify the miRNA panel in the serum of NSCLC patients with different EGFR mutation status [26]. They revealed that the expressions of serum miR-21 and miR-10b were much higher in patients with EGFR mutation than those without mutation. In addition, patients with up-regulated miR-21 expression had a shorter OS time, suggesting that miR-21 expression may be a reliable predictor for gefitinib-treatment efficacy. Furthermore, the levels of miR-10b expression in progressive patients were significantly higher than those in non-metastatic patients. These results suggested that the expression of circulating miRNAs differed between patients with positive and negative EGFR mutation status and the levels of circulating miRNAs are associated with response to EGFR-TKI therapy.

Another study, which aimed to investigate the role of miR-21 in the acquired resistance to EGFR-TKIs, showed that miR-21 can mediate EGFR-TKI resistance by down-regulating PTEN and PDCD4 and activating the PI3K/Akt pathway [25]. Besides, it revealed that the serum miR-21 expression in NSCLC patients receiving EGFR-TKIs was much higher at the time of acquired resistance than that at baseline. These evidences indicated that circulating miR-21 might act as an available biomarker to monitor the acquired resistance to EGFR-TKIs.

The above available evidences suggested that serum miR-21 and miR-10b were helpful for monitoring EGFR-TKI treatment. Future data may update these results and validate the practical utility of circulating miRNAs in NSCLC.

## Proteomic biomarkers

Similar to other high-throughput technologies, proteomics are able to detect multiple proteins that are expressed as a cause or consequence of ongoing diseases [68]. Proteomic profiles of various specimens obtained from cancer patients are expected to increase our understanding of tumor pathogenesis and cancer therapy. In the management of NSCLC, proteomic profiles have been shown to be useful in disease detection, diagnosis, treatment, and prognostication [69]. Recent studies revealed that a simple serum-based proteomic test had a great potential for selecting NSCLC patients to receive EGFR-TKI treatment [28–31].

Taguchi and his colleagues reported a mass spectrometry serum test which was able to identify a subset of NSCLC patients with better outcomes after EGFR-TKI therapy in 2007 [28]. Then this test was commercialized under the name of VeriStrat and applied in multiple clinical trials. This novel test used the integrated intensities of eight mass spectral peaks and classified patients into VeriStrat “good” and VeriStrat “poor” statuses. Several subsequent studies also demonstrated that VeriStrat “good” patients could benefit more from EGFR-TKI therapy than “poor” ones [29–31, 70, 71]. Additionally, a large meta-analysis based on these studies confirmed that the VeriStrat “good” status predicted a better clinical outcome with a pooled HR of 0.40 (95 % CI, 0.32 to 0.49; *p* = 0.000) for OS and 0.49 (95 % CI, 0.39 to 0.60; *p* = 0.000) for PFS [72]. Furthermore, the original study has tested the concordance of mass spectrometry generated by two independent institutions [28]. Their results indicated that the VeriStrat test had an outstanding reproducibility. Thus, the VeriStrat test might be a potential tool to predict the clinical outcome of NSCLC patients after treatment with EGFR-TKIs.

The PROSE trial, the first prospective, randomized proteomic stratified phase III study, was designed to evaluate the predictive value of the VeriStrat test on the outcome in advanced NSCLC patients treated with either EGFR-TKIs or standard chemotherapy [73]. Information provided by this trial suggested that this proteomic test is predictive of OS benefit for erlotinib and chemotherapy. Patients with a VeriStrat “poor” status had worse survival on erlotinib than on chemotherapy (HR, 1.72; 95 % CI, 1.08–2.74; *p* = 0.022). Chemotherapy is likely to result in improved outcomes for patients with a VeriStrat “poor” status. This trial complemented the results of previous retrospective studies and helped to identify the subgroup of NSCLC patients who may not benefit from EGFR-TKIs.

In this emerging field, the number of studies is relatively limited but growing. Another phase III study (EMPHASIS) was opened for advanced squamous cell NSCLC (Clinicaltrials.gov identifier: NCT01652469). Patients with relapsed squamous cell lung cancer are assigned to two groups (VeriStrat “good” and VeriStrat “poor”) and then randomized between EGFR-TKI (erlotinib) and chemotherapy (docetaxel). One of its goals is to explore the predictive and prognostic ability of the VeriStrat test signature. In addition, a clinical trial that aims to measure whether VeriStrat could be a biomarker of benefit from treatment with standard chemotherapy regimens in first-line NSCLC patients is currently recruiting participants (Clinicaltrials.gov identifier: NCT02055144). These ongoing trials may prospectively validate the predictive role of this serum-based proteomic test in NSCLC patients.

## Circulating tumor cells

Circulating tumor cells (CTCs), which are disseminated from tumor tissues, have the potential to complement tumor tissues for tumor characterization and cancer-treatment monitoring [74]. Various kinds of genetic abnormalities, such as KRAS mutation [75], EGFR mutation [32, 33], and ALK rearrangement [76–78], have been identified in CTCs from NSCLC patients. Moreover, CTCs represent promising materials for EGFR-TKI-treatment monitoring.

### Detection of EGFR gene mutations

The EGFR gene mutation profiles of CTCs in NSCLC have been analyzed by several studies. Recently, a study investigated the EGFR mutations in CTCs by coupling the CellSearch System with next-generation sequencing (NGS) [32]. In 37 advanced NSCLC patients harboring activating EGFR mutations in tumor tissues, 31 cases were identified to have the EGFR mutations in CTCs. Additionally, 94 % (29/31) of the mutation types detected by NGS on CTCs were in accordance with that found in matched tumor tissues. These data suggested that CTCs represented a suitable source for detection of EGFR mutations. Another study developed an available method to detect EGFR mutations in single CTC [33]. The laser cell microdissection (LCM) technique was used to isolate individual CTCs. Then it was followed by the whole-genome amplification of DNA for exon 19 deletion and L858R and T790M mutation detection. Ninety-five percent (19/20) of the single CTC underwent PCR amplicons for at least one of the three mutation sites. The amplification success rates were 55 % (11/20) for exon 19 deletion, 45 % (9/20) for T790M, and 85 % (17/20) for L858R. These results suggested that detecting EGFR mutations in CTCs was feasible. CTCs may be available materials for the detection of EGFR mutations in NSCLC.

### Monitoring EGFR-TKI-treatment efficacy

As mentioned above, the dynamic changes in ctDNA-based EGFR status are able to predict the benefit of EGFR-TKI treatment [24]. Similarly, EGFR mutation status in CTCs can be another biomarker for monitoring EGFR-TKI-treatment efficacy. Breitenbuecher et al. adopted an assay based on real-time polymerase chain reaction and melting curve analysis to detect activating EGFR mutations in CTCs [34]. All CTCs in baseline blood samples from eight NSCLC patients were found to harbor the EGFR mutations that corresponded with tumor tissues. During treatment, sequential samples from four of eight patients turned “negative” for EGFR mutations. These four patients who “cleared” EGFR-mutant CTCs achieved disease control, with three partial responses (PRs) and one stable disease (SD). In the remaining four patients with persisting EGFR-mutant CTCs, only one had PR. In addition, patients who had “cleared” EGFR-mutant CTCs showed a significantly longer recurrence-free time when compared with patients whose EGFR mutations in CTCs remained “positive” (median time, 355 versus 116 days). With the follow-up time extending, all four patients with “cleared” EGFR-mutant CTCs developed progressive disease. Interestingly, EGFR mutation status in CTCs from these four patients returned to “positive” prior to clinical progression. Thus, an increase in EGFR-mutant CTC counts might be an early indicator for disease relapse and EGFR-TKI resistance. Moreover, changes in CTC levels were also capable of predicting the outcome of EGFR-TKI treatment. Punnoose et al. performed a study to evaluate the utility of CTCs in NSCLC patients treated with pertuzumab and erlotinib [37]. Their results revealed that decreased CTC counts upon treatment were associated with better response and longer PFS. Thus, CTCs are effective to monitor EGFR-TKI-treatment efficacy in NSCLC patients.

### Tracking emergence of EGFR-TKI resistance

DNA extracted from CTCs, like other sources of DNA, has been shown to be an effective material for the detection of the T790M mutation of EGFR in NSCLC. Maheswaran et al. performed an EGFR mutation analysis on CTCs in NSCLC patients treated with EGFR-TKIs [79]. T790M mutation was detected in CTCs from 9 of 14 patients (64 %) who had clinical progression. This finding was consistent with the reported prevalence of T790M (about 50 %) [80]. Hence, CTCs collected during treatment might provide a potential alternative for EGFR-TKI-resistance analysis. Future large-scaled prospective studies may verify the practical utility of CTCs in EGFR-TKI-treatment monitoring.

## Conclusions

Non-invasive approaches, usually based on serum or plasma samples, are showing great potential to monitor EGFR-TKI treatment in NSCLC (Fig. 1 and Table 2). ctDNA has a high degree of specificity to detect EGFR mutations. Moreover, ctDNA is capable of monitoring disease progression during EGFR-TKI treatment. Certain serum microRNAs, such as miR-21 and miR-10b, can effectively predict the EGFR-TKI-treatment efficacy and resistance. The VeriStrat test has been proved to be helpful for selecting NSCLC patients to receive EGFR-TKIs. CTCs are feasible for EGFR mutation analysis and represent promising materials to monitor EGFR-TKI treatment. Thus, these liquid specimens can complement with tumor tissues and help to guide EGFR-TKI therapy in NSCLC. As numerous high-throughput detection techniques are being developed extensively, liquid samples obtained by non-invasive approaches possess great potential to be valuable materials applied for guiding individual treatment. However, several hurdles should be overcome before applying these approaches into routine clinical practice (Table 2). One of the most important issues is the lack of consensus in technical approaches. Future standard and universal techniques for these non-invasive approaches may validate their applications in monitoring EGFR-TKI treatment in NSCLC.

**Table 2:** Summary of non-invasive approaches to monitor EGFR-TKI treatment in NSCLC patients

Non-invasive approaches	Methods	Sensitivity	Specificity	Advantages	Disadvantages	Cost
ctDNA	PCR-based techniques; DNA sequencing	Moderate	High	Feasible on small-amount samples; suitable for detecting specific genes; high reproducibility	Normalization problems	Low
MicroRNAs	RT-PCR-based techniques	Moderate	High	Feasible on small-amount samples; rapid and low cost; high reproducibility	Normalization problems; indirect evidence; few correlative studies	Low
Proteomic biomarkers	Mass spectrometry	High	Moderate	Feasible on small-amount samples; suitable for detecting specific proteins	Normalization problems; complex tumor proteins profile; indirect evidence	Moderate
CTCs	Cell enrichment techniques + PCR-based techniques or DNA sequencing	High	High	Able to analyze distinct cell subpopulations; suitable for detecting specific genes	Low frequency; dependent on capture techniques; normalization problems	High

*ctDNA* circulating cell-free tumor DNA, *PCR* polymerase chain reaction, *RT-PCR* reverse transcription-polymerase chain reaction, *CTCs* circulating tumor cells
